# MiTF links Erk1/2 kinase and p21^CIP1/WAF1 ^activation after UVC radiation in normal human melanocytes and melanoma cells

**DOI:** 10.1186/1476-4598-9-214

**Published:** 2010-08-11

**Authors:** Feng Liu, Amarinder Singh, Zhen Yang, Angela Garcia, Yu Kong, Frank L Meyskens

**Affiliations:** 1 Department of Medicine, University of California-Irvine School of Medicine, Orange, CA 92868, USA; 2 Chao Family Comprehensive Cancer Center, University of California-Irvine School of Medicine, Orange, CA 92868, USA; 3 Department of Biological Sciences, University of California-Irvine, Irvine, CA 92617, USA; 4 Shandong Provincial Hospital, Jingwu Road, Shandong Province, 250021, China; 5 Department of Chemistry, School of Life Science, Xi'an Jiao Tong University, Xi'an, Shaanxi Province, 710049, China

## Abstract

As a survival factor for melanocytes lineage cells, MiTF plays multiple roles in development and melanomagenesis. What role MiTF plays in the DNA damage response is currently unknown. In this report we observed that MiTF was phosphorylated at serine 73 after UVC radiation, which was followed by proteasome-mediated degradation. Unlike after c-Kit stimulation, inhibiting p90RSK-1 did not abolish the band shift of MiTF protein, nor did it abolish the UVC-mediated MiTF degradation, suggesting that phosphorylation on serine 73 by Erk1/2 is a key event after UVC. Furthermore, the MiTF-S73A mutant (Serine 73 changed to Alanine via site-directed mutagenesis) was unable to degrade and was continuously expressed after UVC exposure. Compared to A375 melanoma cells expressing wild-type MiTF (MiTF-WT), cells expressing MiTF-S73A mutant showed less p21^WAF1/CIP1 ^accumulation and a delayed p21^WAF1/CIP1 ^recovery after UVC. Consequently, cells expressing MiTF-WT showed a temporary G1 arrest after UVC, but cells expressing MiTF-S73A mutant or lack of MiTF expression did not. Finally, cell lines with high levels of MiTF expression showed higher resistance to UVC-induced cell death than those with low-level MiTF. These data suggest that MiTF mediates a survival signal linking Erk1/2 activation and p21^WAF1/CIP1 ^regulation via phosphorylation on serine 73, which facilitates cell cycle arrest. In addition, our data also showed that exposure to different wavelengths of UV light elicited different signal pathways involving MiTF.

## Background

MiTF plays a critical role in melanocyte lineage differentiation and survival [1], as well as melanomagenesis [2]. The *MiTF *gene is amplified in about 20% of melanomas and is capable of transforming normal melanocytes in certain genetic environments, therefore it has been suggested that MiTF can function as an oncogene [3,4]. However, re-expression of MiTF in BRAF-expressing human melanocytes inhibited cell proliferation, suggesting that MiTF represses cell cycle progression [5]. This is consistent with reports showing that MiTF activates the cyclin-dependent kinase inhibitors p21^WAF1/CIP1 ^and p16^INK4A ^[6,7]. More and more evidence indicates that MiTF plays multiple roles in melanomagenesis including stimulating angiogenesis via activating Hif1α [8], enhancing cell proliferation via activating transcription of Bcl-2 and CDK2 [9,10], preventing apoptosis via activating melanoma inhibitor of apoptosis (ML-IAP) [11], inhibiting invasion via activating DIAPH-1 [12], and promoting survival after elevation of cellular reactive oxygen species via activating Ape/Ref-1 [13]. A recent study using mouse melanocytes with various MiTF doses indicated that MiTF dose was a primary determinant for murine melanocytes survival after UVR [14]; however, the mechanism(s) by which this occurred was not clear.

A genetic hallmark of human melanoma is mutually exclusive mutations of BRAF and NRAS, which are found in more than 90% of tumors [15]. Oncogenic BRAF or NRAS mutations activate cell proliferation pathway through downstream mitogen-activated kinases Mek1/2 and extracellular signal-regulated kinase (Erk1/2) [16]. BRAF or NRAS activation leads to Mek1/2 activation which in turn activates Erk1/2 which directly phosphorylates MiTF at serine 73 [17,18]. Activated Erk1/2 can further activate its downstream kinase p90-RSK1 which can also phosphorylate MiTF at serine 409 [19]. Phosphorylation at both sites triggered by c-Kit stimulation leads to a signal cascade for pigment cell development [19]. This dual phosphorylation results in a transient increase of MiTF trans-activation activity and a subsequent degradation; however, the biological consequence of this transient activation and degradation is not clear. Recently *in vivo *studies indicated that mutation at serine 73 completely rescued mouse coat color [20], suggesting this mutation may have other functions than melanocyte development, among which participating in the DNA damage response is one of the possibilities [21]. Whether MiTF plays a role in DNA damage response has not been previously reported and is the subject of this study.

In this study, we report that the DNA damaging agent UVC radiation leads to Erk1/2 mediated phosphorylation of MiTF at serine 73, which in turn leads to proteasome-mediated MiTF degradation. Erk1/2 phosphorylation of MiTF played a critical role in activating p21^WAF1/CIP1 ^transcription and a temporary G1 cell cycle arrest, which enhanced cell survival after UVC radiation. These results suggest a novel function of MiTF in linking Erk1/2 activation and p21^WAF1/CIP1 ^regulation after UVC radiation in normal human melanocytes and melanoma cells.

## Results

### MiTF is phosphorylated and transiently degraded after UVC in NHMs and some melanoma cells

**Figure 1:**
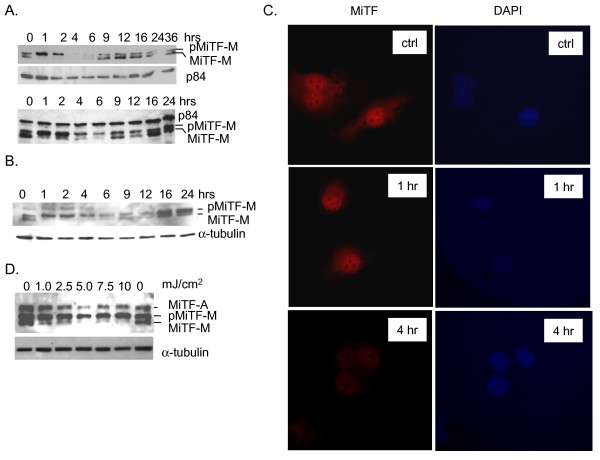
**MiTF was phosphorylated and degraded after UVC**. A, Normal human melanocytes were exposed to 3 mJ/cm^2 ^of UVC and collected at various time points for western blot analysis. NHMs from a white individual (top panel) and a black individual (bottom panel) showed a similar response. B, human melanoma c83-2C cells were subjected to 3 mJ/cm^2 ^of UVC and collected for western blot at the indicated time points. C, c83-2C cells were treated with UVC and fixed at the indicated time and subjected to immunofluorescence detection of MiTF protein. DAPI staining was used to show nucleus. D, dose-dependent phosphorylation of MiTF in c83-2C cells. In western blot, either p84 or α-tubulin was used as a loading control.

To examine whether MiTF plays a role in DNA damage response, two normal human melanocyte (NHM) cell lines were exposed to potent DNA damaging agent UVC (3 mJ/cm^2^) and allowed them to recover for various periods of time. As shown in Fig 1A, MiTF at baseline was detected as a doublet band on western blot: the lower band represented unphosphorylated and the top band the phosphorylated form of MiTF [19]. One hour after UVC, all the MiTF was shifted to the top band (Fig 1A). The phosphorylation continued for 2 hours after UVC, followed by a decrease of MiTF protein (both forms) at 4 and 6 hours. After that, MiTF protein started to recover 9 hours post radiation and nearly completely recovered to its pre-treatment levels 12 to 24 hours after UVC (Fig 1A).

The two NHMs were isolated from neonatal foreskin of a Caucasian (top) and an African black baby (bottom) respectively. There was no significant difference in their response to UVC. A similar response was observed in c83-2C melanoma cells (Fig 1B). MiTF degradation was further confirmed by immunofluorescence (Fig 1C). c83-2C cells were exposed to UVC and fixed for immunofluorescence staining at various time points. Consistent with its nuclear localization, the fluorescence signal for MiTF was mainly observed in nuclei (Fig 1C). However, no specific foci were observed, nor was there a dramatic re-localization of the protein at 1 hour post radiation, suggesting that phosphorylation of MiTF was not a signal for recruiting DNA repair proteins to DNA-damage sites, nor was it a signal for translocation to cytoplasm. MiTF phosphorylation was examined 1 hour after various doses of UVC radiation; as low as 1 mJ/cm^2 ^of radiation led to MiTF phosphorylation in c83-2C cells (Fig 1D).

### MiTF phosphorylation is via Erk1/2 mitogen-activated protein kinases (MAPK) and is required for its subsequent proteasome-dependent degradation

**Figure 2:**
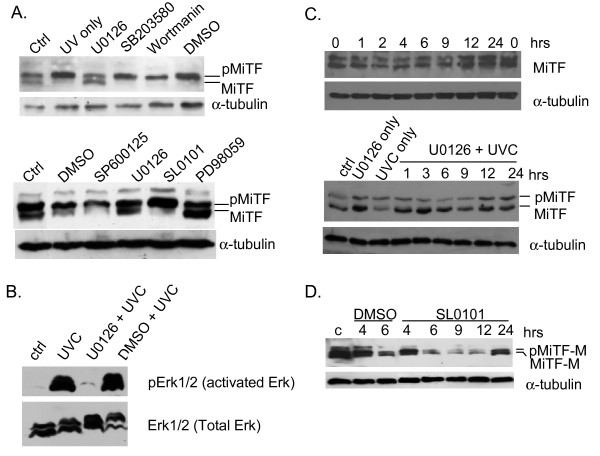
**MiTF was phosphorylated by Erk1/2 kinase after UVC**. A, NHM (top panel) or c83-2C cells (bottom panel) were pre-treated with various kinase inhibitors and then exposed to UVR at 3 mJ/cm^2^, and collected 1 hour post-radiation for western blot analysis. B, UVC-mediated Erk1/2 activation and U0126 effect on Erk1/2 inhibition was examined by western blot. NHM cells were pre-treated with U0126, exposed to UVC and collected for western blot 1 hour post-radiation. C, c83-2C cells (top panel) and Malme-3 M cells (bottom) were pre-treated with U0126 and then subjected to UVR at 3 mJ/cm^2^, and collected at the indicated time points for western blot analysis. D, NHM were pre-treated with p90 RSK-1 inhibitor SL0101 and subjected to UVC, and collected at the indicated time for western blot analysis.

To investigate the upstream signal for MiTF phosphorylation, three kinase inhibitors were incubated with NHMs before they were exposed to UVC (3 mJ/cm^2^): MEK inhibitor U0126 which leads to Erk1/2 inhibition (10 μM), the p38 MAPK inhibitor SB203580 (20 μM), and wortamannin (20 μM), an inhibitor of phosphatidylinositol-3 kinase, Ataxia telangiectasia mutated (ATM) and ATM- and Rad3-related (ATR) kinase. Cells were exposed to UVC (3 mJ/cm^2^) and collected 1 hour later to examine MiTF phosphorylation. As shown in Fig 2A, top panel, among these kinase inhibitors, only U0126 inhibited UVC-mediated MiTF phosphorylation, suggesting that Erk1/2 is the upstream kinase. This observation was further confirmed in c83-2C melanoma cells. The c83-2C cells were pre-treated with U0126 (10 μM), c-Jun N-terminal kinase inhibitor SP600125 (10 μM), RSK1/2 inhibitor SL0101 (70 μM) and another Erk1/2 kinase inhibitor PD98059 ((20 μM), and then exposed to UVC (2.5 mJ/cm^2^) and allowed to recover for 1 hour. Both U0126 and PD98059 inhibited UVC-mediated MiTF phosphorylation, while SP600125 and SL0101 did not (Fig 2A, bottom panel). Erk1/2 activation upon UVC radiation and its inhibition by U0126 was confirmed by western blot using phospho-Erk-specific antibodies (Fig 2B).

Next we examined whether the Erk1/2-mediated phosphorylation was required for MiTF degradation after UVC. Pre-treatment with U0126 in c83-2C cells abolished MiTF phosphorylation, as well as its subsequent degradation (Fig 2C, top panel). A similar result was also observed in Malme-3 M melanoma cells pre-treated with U0126 (Fig 2C, bottom panel). These data suggest that phosphorylation of MiTF by Erk1/2 was necessary for its degradation.

It was previously reported that the c-Kit signal triggered dual-phosphorylation of MiTF, one at serine 73 by Erk2 and the other on serine 409 by Erk1/2 downstream kinase p90 RSK-1. To examine whether UVC also exhibited a similar effect on MiTF through p90 RSK-1, we pre-treated c83-2C cells with RSK-1 inhibitor SL0101 before UVC radiation, MiTF degradation was still observed (Fig 2D), suggesting that p90 RSK-1 phosphorylation of MiTF was not a critical event under this condition, and Erk1/2 was the major kinase for UVC-triggered MiTF phosphorylation and degradation.

### Phosphorylation on serine 73 is responsible for proteasome-mediated MiTF degradation

**Figure 3:**
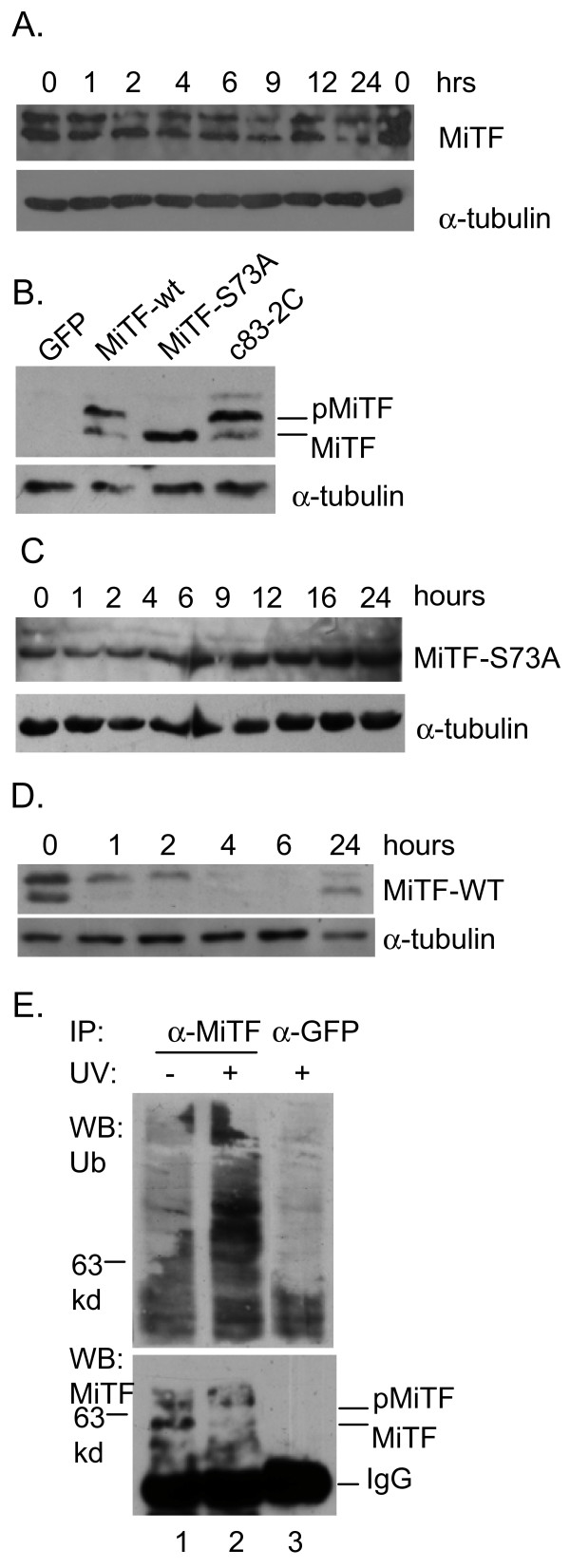
**MiTF phosphorylation on serine 73 by Erk1/2 kinase was required for its proteasome-mediated degradation after UVR**. A, c83-2C cells were exposed to UVC in the presence of MG132 and collected for western blot analysis at the indicated time points. B, transient expression of MiTF-WT, MiTF-S73A in A375 melanoma cell line. C83-2C cells served as a positive control for MiTF western blot. GFP is the control cells transfected with GFP in the same QCXIP vector that carried MiTF-WT or MiTF-S73A coding sequence. C, Cells expressing MiTF-S73A were exposed to UVC and collected for western blot analysis. D, Cells expressing MiTF-WT were exposed to UVC and collected for western blot analysis. E, MiTF was poly-ubiquitinated after UVC radiation. NHMs were exposed to UVC (3 mJ/cm^2^) and collected for immunoprecipitation by anti-MiTF antibodies (un-irradiated cells and anti-GFP antibodies were used as controls), then probed with anti-ubiquitin antibodies (top panel). The membrane was stripped and blotted with anti-MiTF antibodies for a loading control (bottom). The IgG label indicates antibody heavy chain of IgG proteins.

To confirm that MiTF degradation is mediated by proteasome pathway, c83-2C cells were treated with MG132, a proteasome inhibitor and then exposed to UVC. MiTF exhibited an unchanged expression under these conditions (Fig 3A).

Next we expressed MiTF-WT and MiTF-S73A in MiTF-negative A375 melanoma cells, and examined their accumulation after UVC. As shown in Fig 3B, MiTF-WT showed on western blot as a doublet band, MiTF-S73A, on the other hand, exhibited a single band that corresponded to the faster moving band. MiTF-S73A did not show any band shift nor degradation after UVC (Fig 3C), while MiTF-WT was phosphorylated and degraded (Fig 3D). To investigate whether poly-ubiquitination is involved in MiTF regulation after UVC radiation, NHMs were exposed to 3 mJ/cm^2 ^of UVC and then collected 2 hours later for immunoprecipitation. As shown in Fig 3E, UVC dramatically enhanced poly-ubiquitination of MiTF protein (compare Lanes 1 and 2). Anti-GFP antibody was used as a negative control for anti-MiTF antibody (Lane 3). Taken together, these results suggest that Erk1/2-mediated MiTF phosphorylation on serine 73 is required for MiTF degradation after UVC. These results are consistent with previous observation that phosphorylation on serine 73 is essential for MiTF poly-ubiquitination and degradation [22].

### Expression of MiTF-WT led to a temporary G1 arrest and enhanced cell survival in A375 cells but expression of MiTF-S73A did not

**Figure 4:**
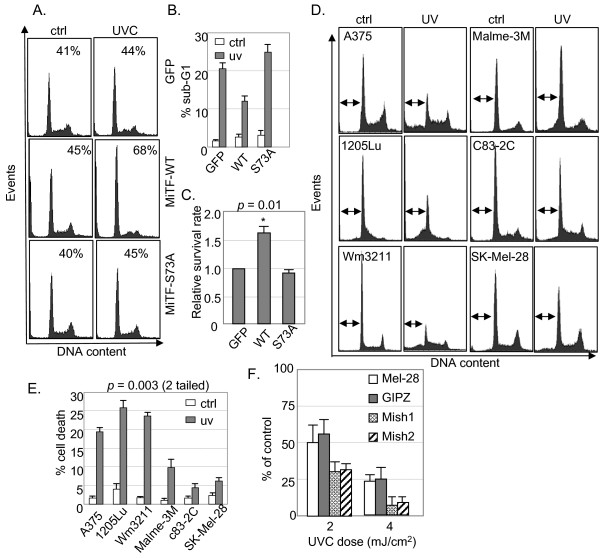
**Expression of MiTF-WT enabled a temporary G1 arrest for improving cell survival after UVR**. A, A375 cells were transfected with GFP, MiTF-WT or MiTF-S73A and then exposed to UVC at 3 mJ/cm^2^, and fixed 8 hours later for FACS analysis after Propidium Iodide staining. B, sub-G1 population of cells treated in A were calculated by FACS analysis and graphed 24 hours after UVC treatment. C, cells in A were seeded and exposed to UVC, then incubated for colony formation assay. Colonies formed 2 weeks after were counted, normalized to that in GFP-expressing cells and graphed. D, A375, WM3211, 1205Lu, Malme-3 M, SK-Mel-28 and c83-2C cells were exposed to UVC at 3 mJ/cm^2^, and cells were then collected 24 hours later for FACS analysis. E, Percentage of cell death before and after UVC were calculated and graphed F, knockdown of MiTF decreased cell survival after UVC. MiTF was knocked down by Mish1 and Mish2 shRNA (see Fig 5E) and exposed to 3 mJ/cm^2 ^of UVC. Colony formation was analyzed about 2 weeks post-radiation.

Cells normally undergo cell cycle arrest after UVC exposure to allow enough time for DNA damage repair [23]. To investigate the role of MiTF in UVC-mediated DNA damage response and cell cycle control, A375 cells which carry a wild-type p53 gene were transfected with QCXIP-GFP (control vector), QCXIP-MiTF-WT or QCXIP-MiTF-S73A and then exposed to UVR (3 mJ/cm^2^). Cell cycle distribution was analyzed by fluorescence-activated cell sorting at various time points after staining with Propidium Iodide (PI). About 40% of cells were in G1 phase when un-irradiated in all three groups. Eight hours after UVR, G1 population in MiTF-WT-expressing cells increased to 68% (Fig 4A), while there were no significant changes in cells expressing MiTF-S73A or GFP. At 24 hours post radiation, the G1 population decreased significantly in all three groups of cells due to cell death (data not shown). Sub-G1 population was then quantified. 21.4% of sub-G1 cells were present in control cells expressing GFP, while only 12.1% of sub-G1 cells were found in cells expressing MiTF-WT (Fig 4B). In cells expressing MiTF-S73A, the sub-G1 population was 25.7%, more than 2 fold higher than that in MiTF-WT-expressing cells and close to what was observed in control GFP cells (Fig 4B).

The above results suggested that expression of MiTF-WT caused a temporary G1 arrest after UVC, which enhanced cell survival. To further confirm this observation, colony formation assay was used to measure cell survival rate after UVC. A375 cells were again transfected with QCXIP-GFP, QCXIP-MiTF-WT or QCXIP-MiTF-S73A and were irradiated with 3 mJ/cm^2 ^of UVC 24 hours after transfection. Colonies were counted 2 weeks later. The relative survival rates were normalized to that of GFP-expressing control cells and the results are shown in Fig 4C. MiTF-WT increased cell survival after UVR, but MiTF-S73A did not.

### MiTF-negative melanoma cells are more sensitive to UVC

**Figure 5:**
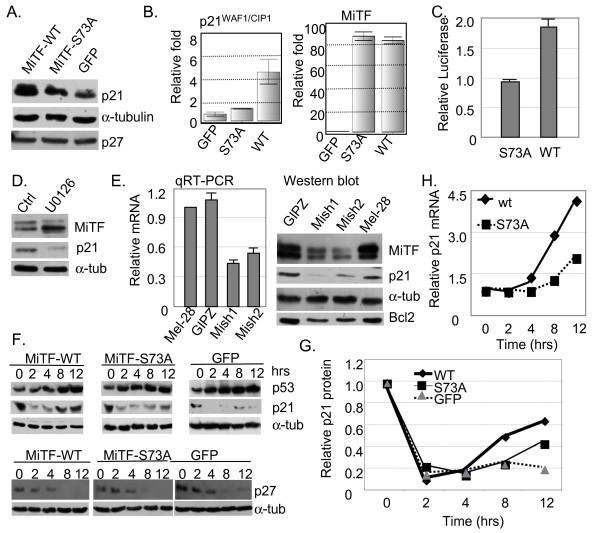
**MiTF-S73A is less potent in activating p21^WAF1/CIP1 ^transcription**. A, p21^WAF1/CIP1 ^and p27^KIP1 ^protein accumulation in A375 cells expressing MiTF-WT, MiTF-S73A and GFP were analyzed by western blot. B, transcripts of p21^WAF1/CIP1 ^and MiTF were analyzed by qRT-PCR in the above cells, α-tubulin was the reference for both genes. C, p21^WAF1/CIP1 ^promoter reporter analysis in cells co-transcfected with MiTF-WT or MiTF-S73A mutant constructs. D, p21^WAF1/CIP1 ^protein accumulation decreased when MiTF phosphorylation was inhibited. NHMs were treated with 20 μM of U0126 for 24 hours and collected for western blot analysis. E, knockdown MiTF led to decreased p21^WAF1/CIP1 ^expression. Quantitative RT-PCR (left) and western blot (right) analysis of p21^WAF1/CIP1 ^expression in control SK-Mel-28 cells (Mel-28), cells transduced with empty lentivirus vector pGIPZ (GIPZ), and cells transduced with lentivirus carrying MiTF shRNA constructs (Mish1 and Mish2). Again α-tubulin was used as a loading control. F, top: p27^KIP1 ^protein accumulation after UVC in A375 cells expressing MiTF-WT, MiTF-S73A and GFP; bottom: p21^WAF1/CIP1 ^protein accumulation after UVC in A375 cells expressing MiTF-WT, MiTF-S73A and GFP. The p53 served as a positive control for UVC radiation, α-tubulin served as a loading control. The western was repeated three times and a representative blot is shown; G, the p21 protein levels in the western blot were quantified by a densitometry, normalized to α-tubulin levels and then normalized to that in cells without irradiation and graphed. H, qRT-PCR analysis of p21^WAF1/CIP1 ^mRNA accumulation after UVC in A375 cells expressing MiTF-WT or MiTF-S73A.

To investigate whether MiTF confers to a survival advantage in other melanoma cell lines, we exposed different melanoma cell lines with different MiTF accumulation levels to 3 mJ/cm^2 ^of UVC and examined the cell survival 24 hours later by Propidium Iodide staining and FACS analysis. As shown in Fig 4D, three melanoma cell lines (A375, 1205Lu and WM3211) which accumulated undetectable MiTF protein [13] showed higher cell death (19% to 26%) as compared to three MiTF-positive melanoma cell lines (Malme-3 M, SK-Mel-28 and c83-2C) (4% to 10%) (Fig 4D and 4E). The difference between these two groups was significant (two tailed p value from an unpaired t test is 0.003). To further confirm that MiTF plays a key role in cell survival after UVC radiation, MiTF was knocked down in SK-Mel-28 melanoma cell line by 2 different shRNA constructs Mish1 and Mish2 (Fig 5E); cells were exposed to 2 and 4 mJ/cm^2 ^of UVC, and colonies were counted 2 weeks later. The results indicated that Mish1 and Mish2 transduced cells showed decreased colony formation after UVC as compared to control parental SK-Mel-28, as well as SK-Mel-28 cells transduced with pGIPZ empty vector (Fig 4F).

### MiTF participates in G1 arrest via its regulation of p21^WAF1/CIP1^

Because p16^INK4A ^is often lost in melanoma cells, we examined accumulation of CDK inhibitors p21^WAF1/CIP1 ^and p27^KIP1^, both of which are downstream of MiTF. MiTF directly activates p21^WAF1/CIP1 ^expression and indirectly activates p27 [6,12]. The basal level of p27^KIP1 ^was not significantly altered in these three groups of cells (Fig 5A). However, p21^WAF1/CIP1 ^level was elevated in cells expressing MiTF-WT as compared to cells expressing MiTF-S73A, which showed a slightly elevated level of p21^WAF1/CIP1 ^as compared to cells expressing GFP (Fig 5A).

To confirm that the regulation of p21^WAF1/CIP1 ^by MiTF was indeed via transcriptional regulation, mRNA from A375 cells expressing MiTF-WT, MiTF-S73A and GFP was isolated and p21^WAF1/CIP1 ^mRNA level determined by quantitative RT-PCR. As shown in Fig 5B, MiTF-WT increased p21^WAF1/CIP1 ^mRNA to about 5 fold that in control GFP-expressing cells, while MiTF-S73A also increased p21^WAF1/CIP1 ^mRNA to about 2 fold of that in control cells. MiTF expression levels were also examined in these cells by qRT-PCR. The control A375 GFP cells expressed very low levels of MiTF, nearly undetectable, which is consistent with our previous observation that no MiTF protein was detectable in A375 cells. In cells transfected with either MiTF-WT or MiTF-S73A constructs the mRNA of MiTF accumulated to approximately 90 fold that in control cells. To further confirm that this regulation is via differential transcriptional activities on the p21^WAF1/CIP1 ^promoter, MiTF-WT or MiTF-S73A constructs were co-transfected with p21^WAF1/CIP1 ^promoter-luciferase reporter plasmid. We observed that expression of MiTF-WT led to about 2 fold of p21^WAF1/CIP1 ^promoter activity as compared to expression of MiTF-S73A mutant (Fig 5C). Further more, treating the NHMs with U0126 (20 μM) caused a decrease on MiTF phosphorylation, which was concomitant with reduced p21^WAF1/CIP1 ^protein levels (Fig 5D). To further confirm regulation of p21^WAF1/CIP1 ^by MiTF, MiTF was knocked down in SK-Mel-28 cells by lentivirus mediated shRNA Mish1 and Mish2 (2 different shRNA constructs). As shown in Fig 5E, both shRNA knocked down MiTF to about 30% of its original protein levels (Fig 5E, right panel), the control lentivirus vector GIPZ did not affect MiTF expression. Both p21^WAF1/CIP1 ^mRNA and protein levels decreased when MiTF was knocked down (Fig 5E). A known MiTF target Bcl2 protein accumulation was also reduced in Mish1 and Mish2 transduced cells (Fig 5E), which may help to explain in part why MiTF knockdown led to decreased cell survival after UVC (Fig 4F).

Next we examined the kinetics of p21^WAF1/CIP1 ^and p27^KIP1 ^after UVC. The p27^KIP1 ^protein showed a rapid degradation after UVC in all cells examined and no difference was observed in these three groups of cells (Fig 5F, top panel), suggesting that p27^KIP1 ^was not responsible for the observed temporary G1 arrest in MiTF-WT-expressing cells. The p21^WAF1/CIP1 ^protein degraded transiently after UVC as previously reported [24] at 2 to 4 hours, and followed by a rapid re-accumulation (Fig 5F, bottom panel). In cells expressing MiTF-WT protein, p21^WAF1/CIP1 ^degraded to less than 20% of its original level 2 to 4 hours post-UVC and recovered to about 50% at 8 hour, over 60% at 12 hour. In cells expressing MiTF-S73A protein, p21^WAF1/CIP1 ^also degraded 2 to 4 hours post UVC; however, at 8 and 12 hour post radiation, it remained at 25% and 42% of that in untreated cells, respectively. Note that the p21^WAF1/CIP1 ^level in MiTF-S73A-expressing cells was already lower than that in MiTF-WT cells. This slower recovery of p21^WAF1/CIP1 ^may also result from less effective activation of p21^WAF1/CIP1 ^by MiTF-S73A mutants. The p21^WAF1/CIP1 ^protein level showed a similar slower recovery in control cells expressing GFP (Fig 5F, bottom panel). The kinetics of p21^WAF1/CIP1 ^protein levels from these western blots were quantified by a densitometer and normalized to the untreated cells, and graphed in Fig 5G. The kinetics of p21^WAF1/CIP1 ^mRNA following UVC radiation was determined by qRT-PCR, normalized to α-tubulin mRNA, and the results are shown in Fig 5H. Interestingly, the mRNA levels of p21^WAF1/CIP1 ^remained basically unchanged during the first 4 hours of recovery, but then it was induced dramatically and rapidly in MiTF-WT cells but to a lesser extend in MiTF-S73A cells (Fig 5H).

### Differential response of MiTF to different wavelengths of UV radiation

**Figure 6:**
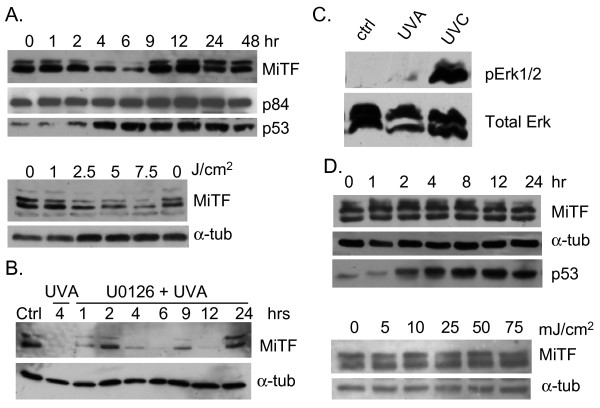
**Distinct responses of MiTF to UVA and UVB radiation**. A, NHMs were exposed to UVA (2.5 J/cm^2^) and collected for western blot at the indicated time points (top panel) or 4 hours post-UVA at various doses (bottom panel). The p53 serves as a positive control and p84 or α-tubulin serve as loading controls. B, NHMs were pre-treated with U0126 and then exposed to UVA and collected for western blot analysis at the indicated time points. C, No obvious Erk1/2 activation after UVA radiation. NHM was irradiated with either UVA (2.5 J/cm^2^) or UVC (3 mJ/cm^2^) and collected for western blot analysis 1 hour post-radiation. D, NHMs were exposed to UVB (25 mJ/cm^2^) and collected for western blot at the indicated time points (top panel) or 1 hour post-UVB at various doses (bottom panel). Again the p53 serves as a positive control and α-tubulin serve as a loading control.

Although UVC is a strong carcinogen and elicits a distinct DNA damage response, UVA and UVB are more directly relevant to melanomagenesis. A large amount of data indicates that these different wavelengths of UV radiation each triggers different signaling cascades upon radiation [25]. We examined how MiTF responded to UVA and UVB radiation. After UVA radiation, MiTF was degraded 4 to 6 hours after radiation without a distinct phase of phosphorylation (Fig 6A, top panel). MiTF protein was restored to its pre-radiation level 9 hours after radiation. The p53 protein accumulation increased from 4 hours post-radiation and served as a positive control for the treatment. The bottom panel of Fig 6A shows the dose-dependent degradation of MiTF 4 hours post-radiation. This degradation was not inhibited by U0126 (Fig 6B), suggesting that there were distinct signal transduction pathways involved in MiTF regulation after UVC and UVA radiation. To further understand this difference, we examined Erk1/2 activation 1 hour after UVA radiation. In fact Erk1/2 did not show substantial activation at this time (Fig 6C). In contrast, MiTF did not exhibit any changes in terms of accumulation levels or phosphorylation status after UVB radiation (Fig 6D). 25 mJ/cm^2 ^of UVB did not affect MiTF accumulation or phosphorylation up to 24 hours (Fig 6D, top panel); Up to 75 mJ/cm^2 ^of UVB radiation did not trigger MiTF phosphorylation at 1 hour after radiation (Fig 6D, bottom panel). As a positive control, p53 up-regulation was observed (Fig 6D).

## Discussion

MiTF is a lineage-specific transcription factor; how it is regulated after DNA damage has not been reported, although it was evident that MiTF dose was correlated with cell survival after UVR [14]. Here we show that the action of MiTF was downstream of Erk1/2 kinase and that phosphorylation on serine 73 played a key role in its trans-activation activity on p21^WAF1/CIP1 ^promoter under these conditions. The Erk1/2 phosphorylation led to proteasome-mediated MiTF degradation, which was concomitant with a temporary G1 cell cycle arrest. Although it was previously known that both Erk1/2 and p21^WAF1/CIP1 ^was activated by UVC [26], a direct link between these two factors was not elucidated. Our data suggest that MiTF participates in G1 cell cycle arrest after UVC via Erk1/2 kinase and p21^WAF1/CIP1 ^regulation, and hence provides a direct link between Erk1/2 kinase and p21^WAF1/CIP1 ^activation.

It was previously reported that Erk2 directly phosphorylated MiTF at serine 73 [18], and this phosphorylation occurred under the condition of c-Kit stimulation, which also triggered a second phosphorylation on serine 409 by p90 RSK-1, leading to a transient increase of its trans-activation activity and subsequent proteasome-mediated MiTF degradation [19]. We observed that under UVC stress, inhibition of Mek1/2 kinase activity led to MiTF stabilization while inhibition of p90 RSK-1 activity did not, suggesting that phosphorylation on serine 73 was the key signaling event after UVC. This was further confirmed by MiTF-S73A mutation which was not degraded after UVC. The degradation was inhibited by proteasome inhibitor MG132, suggesting that the signaling pathways via Erk1/2 activation after UVC and after c-Kit stimulation were distinct from each other.

We observed that re-expression of MiTF-WT in the A375 melanoma cell line restored a temporary G1 arrest after UVC, while control cells expressing GFP or MiTF-S73A cells did not, suggesting that degradation of MiTF after UVC may ensure a proper G1 cell cycle arrest and therefore allow DNA repair and enhance cell survival. In fact we observed that cells expressing MiTF-WT showed better overall survival after UVC. Although MiTF-S73A mutant was present constantly after UVC, it was unable to trigger the G1 arrest. As our data shows, part of the reason may be the weak activation on p21^WAF1/CIP1 ^promoter by this mutant. However, it is also possible that there are other downstream genes differentially regulated by MiTF-WT and MiTF-S73A, therefore affecting the cell cycle progression.

The temporary G1 arrest mediated by MiTF-WT seemed to enhance cell survival after UVC, as the cell death was decreased to about half of that in cells expressing MiTF-S73A or control GFP protein. This result was further confirmed in different melanoma cell lines expressing different levels of MiTF. Cell lines with high levels of MiTF accumulation survived better than cells with lower or un-detectable level of MiTF. This result is consistent with a recent finding that MiTF dose was correlated with cell survival after broad-band UV radiation [14].

As a tumor suppressor playing versatile roles in many aspects of cell cycle progression and DNA replication, p21^WAF1/CIP1 ^is subjected to regulation of multiple transcription factors including p53, Rb, c-Myc and MiTF [6,27,28]. While it is well established that p21^WAF1/CIP1 ^inhibits CDK activities and therefore inhibits cell cycle progression, p21^WAF1/CIP1 ^is also important for DNA replication initiation by binding to proliferating cell nuclear antigen (PCNA) [29,30]. Therefore the precise role of p21^WAF1/CIP1 ^in cell cycle progression is more complicated and remains to be clarified. In A375 melanoma cell lines we observed a transient degradation of p21^WAF1/CIP1 ^and then a rapid recovery of this protein 12 hours after UVC. The early degradation event may serve the purpose of releasing PCNA from replication fork [31] and therefore initiating a G1 arrest, and the subsequent recovery may serve the purpose of inhibiting CKD activities for further maintaining the G1 arrest. CDK inhibitor p27^Kip1 ^usually increases when cell cycle is arrested in G1 phase [32], yet in our experiment we observed that p27^Kip1 ^degraded 8 to 12 hours post-UVC radiation. Intriguingly, while p21^WAF1/CIP1 ^was degraded rapidly 2 to 4 hours post-radiation, p27^Kip1 ^maintained a relatively unchanged level (Fig 5F); when p27^Kip1 ^was degraded 8 hours post-radiation, p21^WAF1/CIP1 ^levels started to restore. It seems these two CDK inhibitors are orchestrated to ensure a G1 arrest in MiTF-expressed A375 cells.

**Figure 7:**
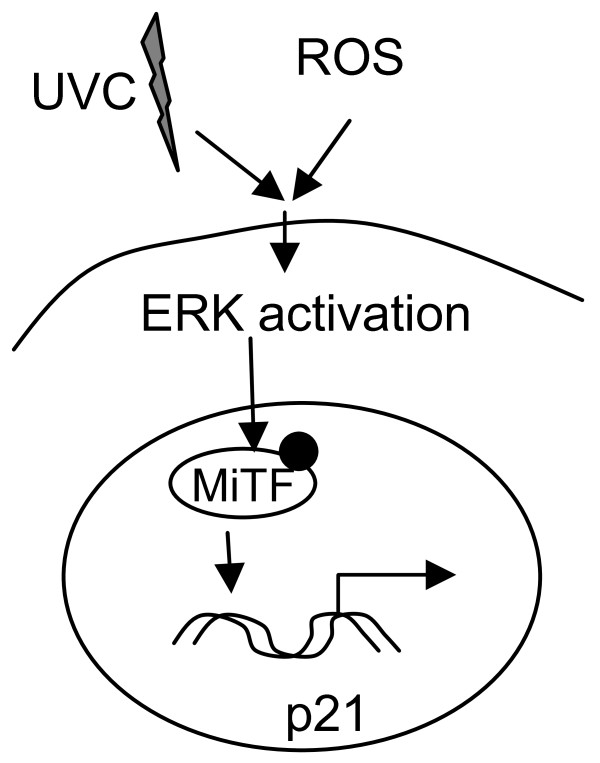
**MiTF mediates signal transduction from Erk1/2 to p21^WAF1/CIP1 ^after UVC and ROS stress**. UVC and ROS trigger Erk1/2 activation, which leads to MiTF phosphorylation and degradation, and enables a temporary G1 cell cycle arrest and subsequent cell survival.

Previously we showed that MiTF was temporarily degraded after elevation of cellular reactive oxygen species levels [13], a process that was also mediated by Erk1/2 kinase. Considering that both UVC and ROS causes similar DNA damages and therefore may employ similar repair pathways [33], the Erk1/2-mediated phosphorylation and degradation of MiTF may reflect a general mechanism of MiTF-mediated survival pathways which is outlined in Fig 7. Upon UVR or ROS stress, MAP kinase is activated which leads to phosphorylation of MiTF on serine 73 and subsequent degradation of MiTF protein. The temporary degradation was correlated with a temporary G1 cell cycle arrest, corresponding with p21^WAF1/CIP1 ^degradation and re-activation, which allows sufficient time for DNA damage repair and ensure of a better cell survival (Fig 7).

In response to UVB radiation, MiTF levels were not changed at the examined dose and time range, nor its phosphorylation status (Fig 6D). However, MiTF was degraded without obvious band shifting after UVA treatment (Fig 6A). Pre-treatment with U0126 also did not prevent MiTF degradation after UVA radiation, suggesting that after UVA MiTF was not phosphorylated by Erk1/2 kinase, nor was the degradation mediated by phosphorylation. These data indicate that signaling pathways after UVA, UVB and UVC are different, which is consistent with previous observations that different wavelengths of UV light trigger different cellular responses [34]. The UVA-MiTF signaling pathway is still under intensive investigation in our laboratory.

## Conclusions

In summary, our data indicated that MiTF played an active role in response to UVC radiation by directly linking Erk1/2 and p21^WAF1/CIP1 ^activation. Erk1/2 kinase is downstream of BRAF and NRAS pathways, which are frequently mutated in human melanomas [35]. Recently it was reported that the MiTF pathway was also frequently mutated in human melanomas [36-38]. Taken together, mutations in these pathways may compromise the cellular defense mechanisms against UV-mediated DNA damage and therefore increase the genome instability, eventually leading to melanomagenesis.

## Methods

### Cell lines and cell culture

Normal human melanocytes were isolated from new-born foreskin followed the procedure by Eisinger and Marco [39], and cultured in MCDB153 medium (Sigma, St. Louis, MO) containing 2% FCS, 0.3% bovine pituitary extract (Cambrex Bio Science Walkersville, Inc., Walkersville, MD), 10 ng/mL 12-*O*-tetradecanoylphorbol-13-acetate (TPA), 2 mmol/L CaCl_2_, 5 μg/mL insulin, and 0.1 mmol/L IBMX (Sigma) (Yang et al.: 2005). Melanoma Malme-3 M cells were cultured in IMDM media containing 20% FBS and 1% penicillin and streptomycin. The c83-2C, A375, SK-Mel-28 or SK-Mel-5 cells were cultured in F10, DMEM, EMEM or AMEM media; each supplied with 5% FBS, 5% new born bovine sera, and 2% penicillin and streptomycin. All cells were kept at 37°C in 5% CO_2 _incubator.

### UV radiation and cell treatment

Cells were grown to about 70% confluence and media was removed completely for UVB and UVC radiation. For UVA radiation, 5 ml of 1× PBS was added to one 10-cm dish of cells and ice cubes were placed next to dishes for absorbing the heat generated by UVA. UVC radiation was performed in a tissue culture hood with genotoxic UVC lamp (peak wavelength 254 nm). UVB radiation was performed in a Stratagen crosslinker with peak wavelength at 312 nm; and UVA radiation was also performed in a Stratagen crosslinker with lamps with peak wavelength at 350 nm. The UV intensity was measured by a radiometer with proper probes. The culture media was returned to cells after radiation and cells were returned to 37°C incubator for recovering. For kinase inhibitor treatment, inhibitors were added into culture media 20 minutes before radiation; cells remained in 37°C incubator during the 20 minutes treatment. Culture media were then removed and cells were exposed to UVR. Fresh media was added into irradiated cells without further washing to leave residue kinase inhibitors in the media.

### DNA constructs and lentivirus transduction

Wild-type MiTF cDNA was cloned into expression vector QCXIP (Clontech, Mountain View, CA) via EcoR I and Apa I sites. MiTF-S73A mutant was a gift from Dr. David Fisher (Harvard Medical School, MA), and was also cloned into QCXIP vector via the same restriction enzyme sites. MiTF-S409A mutant was generated using site-directed mutagenesis kit from Stratagen following the manufacturer's instruction, with the following primers: S409-r, 5'-TCCGTC TCTTCC ATGCTC ATAGCG CTCCTC CGGCTG CTTGTT-3', and S409-f, 5'-AACAAG CAGCCG GAGGAG CGCTAT GAGCAT GGAAGA GAC GGA-3'. All mutations were confirmed by DNA sequencing. The QCXIP-GFP vector was generated by ligating GFP coding sequence from pEGFP-N1 (Nhe I and BamH I fragment) into the BamH I site on QCXIP vector. The p21^WAF1/CIP1 ^promoter construct (pWWP-Luc, containing about 2.4 kb promoter region) was a kind gift from Dr. Wafik El-Deiry (University of Pennsylvania). The Mish1 and Mish2 shRNA plasmids were purchased from Open Biosystems. These plasmids were co-transfected with pMD2G and pSPAX2 plasmids into 293T cells for virus production http://tronolab.epfl.ch/. Transduction was performed in the presence of 10 μg/ml of protamine, using the filtered 293T media as virus source.

### Flow-cytometry and cell cycle analysis

Cells were trypsinized and washed once with 1× PBS, fixed in cold 70% ethanol overnight or until use. Cells were incubated in Propidium Iodide (PI) staining solution in dark for 30 minutes: 50 μg/ml PI, 0.1% sodium citrate, 50 μg/ml RNase A, 0.03% NP-40 in 1× PBS. 10,000 total events were counted for each sample. Cell populations from each phase were calculated according to CellQuest instructions (BD Biosciences).

### Cell lysate and western blot analysis

Cell pellet was lysed in a lysis 250 buffer [40] and quantified by the Bradford protein assay method (Bio-Rad, Richmond, CA). Western blot was performed using antibodies against MiTF C5 plus D5 (MS-773-P, Lab Vision, Fremont, CA), p21 (Santa Cruz), p27 (Santa Cruz), p53 DO-1 (Santa Cruz), p84 (Abcam, Cambridge, MA) and α-tubulin (T9026, Sigma, St. Louis, MO), ubiquitin (ab7780, Abcam). All western blots were repeated at least twice, one representative blot is shown in figures.

### Quantitative Real Time RT-PCR and promoter reporter analysis

One microgram of total RNA isolated from cells was used for first-strand DNA synthesis with random primers. One-twentieth of the total cDNA was subjected to real time PCR amplification in an iCycler iQ5 Real Time PCR Instrument using iQ SYBR Green Supermix from Bio-Rad (170-8882). For the α-tubulin and *p21*^*CIP1/WAF1 *^cDNAs, we used primers AT1 (5'-GCG TGA TGG TGG GCA TGG GTC AG-3') and AT2 (5'-AGG GGG GCC TCG GTC AGC AGC AC-3') and primers p21-f (5'-GAA GAA GGG TAG CTG GGG CT -3') and p21-r (5'-CTC TAA GGT TGG GCA GGG TG -3), respectively. The primers For MiTF were mi6a (5'-CCA ACC GGC ATT TGT TGC TCA-3') and mi2b (5'-GTT GTT GAA GGT GAT GGT GCC-3'). Promoter reporter analysis was carried out using dual luciferase assay system from Promega. Renilla luciferase driven by SV40 early promoter (pSV40-RL) was used as an internal control.

### Immunofluorescence

Cells were seeded on cover slips and treated as indicated, then fixed in 4% formaldehyde solution in 1× PBS at room temperature for 30 minutes. After three washes in 1× PBS, cells were treated with 0.05% Saponin at room temperature. Cells were washed in 1× PBS again for 3 times, and incubated with 10% normal goat serum for 1 hour at room temperature. Cells were further incubated with primary antibody C5 (1:50 dilution in 10% goat serum) at 4°C overnight. After 5 brief wash with 1× PBS plus 0.01% NP-40, cells were incubated with Texas-Red labeled anti-mouse secondary antibody in dark for 1 hour at room temperature. 1 μg/ml DAPI was added into staining solution at the last 10 minutes of incubation for the secondary antibody. Cells were then washed and mounted to a slide for viewing under a Zeiss fluorescence microscope.

### Colony formation assay

Cells were irradiated and then returned to incubator with fresh media. Culture media was changed every three days for 2 weeks. Plates were stained with 0.5% crystal violet solution in 25% methanol. Only colonies with more than 50 cells were counted.

## Competing interests

The authors declare that they have no competing interests.

## Authors' contributions

FL conceived of and designed the study, and carried out most of the experiments and drafted the manuscript. AR carried out the UV-mediated cell survival analysis. AG and YK carried out MiTF shRNA knockdown experiment and colony formation analysis. ZY participated in the western blot experiments. FLM participated in the study design, coordination and direction, and edited the manuscript. All authors read and approved the final manuscript.

## References

[B1] Widlund HR Fisher DE Microphthalamia-associated transcription factor: a critical regulator of pigment cell development and survival Oncogene 2003 22 20 3035 41 10.1038/sj.onc.1206443 12789278

[B2] Mitra D Fisher DE Transcriptional regulation in melanoma Hematol Oncol Clin North Am 2009 23 3 447 65 viii 10.1016/j.hoc.2009.03.003 19464596

[B3] Garraway LA Integrative genomic analyses identify MITF as a lineage survival oncogene amplified in malignant melanoma Nature 2005 436 7047 117 22 10.1038/nature03664 16001072

[B4] Joo A STAT3 and MITF cooperatively induce cellular transformation through upregulation of c-fos expression Oncogene 2004 23 3 726 34 10.1038/sj.onc.1207174 14737107

[B5] Wellbrock C Marais R Elevated expression of MITF counteracts B-RAF-stimulated melanocyte and melanoma cell proliferation J Cell Biol 2005 170 5 703 8 10.1083/jcb.200505059 16129781 PMC2171350

[B6] Carreira S Mitf cooperates with Rb1 and activates p21Cip1 expression to regulate cell cycle progression Nature 2005 433 7027 764 9 10.1038/nature03269 15716956

[B7] Loercher AE MITF links differentiation with cell cycle arrest in melanocytes by transcriptional activation of INK4A J Cell Biol 2005 168 1 35 40 10.1083/jcb.200410115 15623583 PMC2171666

[B8] Busca R Hypoxia inducible factor 1a is a new target of microphthalmia-associated transcription factor (MITF) in melanoma cells Med Sci (Paris) 2006 22 1 10 3 16386209 10.1051/medsci/200622110

[B9] Du J Critical role of CDK2 for melanoma growth linked to its melanocyte-specific transcriptional regulation by MITF Cancer Cell 2004 6 6 565 76 10.1016/j.ccr.2004.10.014 15607961

[B10] McGill GG Bcl2 regulation by the melanocyte master regulator Mitf modulates lineage survival and melanoma cell viability Cell 2002 109 6 707 18 10.1016/S0092-8674(02)00762-6 12086670

[B11] Dynek JN Microphthalmia-associated transcription factor is a critical transcriptional regulator of melanoma inhibitor of apoptosis in melanomas Cancer Res 2008 68 9 3124 32 10.1158/0008-5472.CAN-07-6622 18451137

[B12] Carreira S Mitf regulation of Dia1 controls melanoma proliferation and invasiveness Genes Dev 2006 20 24 3426 39 10.1101/gad.406406 17182868 PMC1698449

[B13] Liu F Fu Y Meyskens FL Jr MiTF regulates cellular response to reactive oxygen species through transcriptional regulation of APE-1/Ref-1 J Invest Dermatol 2009 129 2 422 31 10.1038/jid.2008.255 18971960 PMC4321967

[B14] Hornyak TJ Mitf dosage as a primary determinant of melanocyte survival after ultraviolet irradiation Pigment Cell Melanoma Res 2009 22 3 307 18 10.1111/j.1755-148X.2009.00551.x 19192212 PMC6980044

[B15] Omholt K NRAS and BRAF mutations arise early during melanoma pathogenesis and are preserved throughout tumor progression Clin Cancer Res 2003 9 17 6483 8 14695152

[B16] Meier F The RAS/RAF/MEK/ERK and PI3K/AKT signaling pathways present molecular targets for the effective treatment of advanced melanoma Front Biosci 2005 10 2986 3001 10.2741/1755 15970553

[B17] Molina DM Grewal S Bardwell L Characterization of an ERK-binding domain in microphthalmia-associated transcription factor and differential inhibition of ERK2-mediated substrate phosphorylation J Biol Chem 2005 280 51 42051 60 10.1074/jbc.M510590200 16246839 PMC3017498

[B18] Hemesath TJ MAP kinase links the transcription factor Microphthalmia to c-Kit signalling in melanocytes Nature 1998 391 6664 298 301 10.1038/34681 9440696

[B19] Wu M c-Kit triggers dual phosphorylations, which couple activation and degradation of the essential melanocyte factor Mi Genes Dev 2000 14 3 301 12 10673502 PMC316361

[B20] Bauer GL The role of MITF phosphorylation sites during coat color and eye development in mice analyzed by bacterial artificial chromosome transgene rescue Genetics 2009 183 2 581 94 10.1534/genetics.109.103945 19635938 PMC2766318

[B21] Bertolotto C Ballotti R Functional role of MITF phosphorylation. In vivo veritas? Pigment Cell Melanoma Res 2009 22 6 703 4 10.1111/j.1755-148X.2009.00623.x 19694979

[B22] Xu W Regulation of microphthalmia-associated transcription factor MITF protein levels by association with the ubiquitin-conjugating enzyme hUBC9 Exp Cell Res 2000 255 2 135 43 10.1006/excr.2000.4803 10694430

[B23] Sanchez Y Elledge SJ Stopped for repairs Bioessays 1995 17 6 545 8 10.1002/bies.950170611 7575496

[B24] Bendjennat M UV irradiation triggers ubiquitin-dependent degradation of p21(WAF1) to promote DNA repair Cell 2003 114 5 599 610 10.1016/j.cell.2003.08.001 13678583

[B25] Molho-Pessach V Lotem M Ultraviolet radiation and cutaneous carcinogenesis Curr Probl Dermatol 2007 35 14 27 full_text 17641487 10.1159/000106407

[B26] Bode AM Dong Z Mitogen-activated protein kinase activation in UV-induced signal transduction Sci STKE 2003 2003 167 RE2 10.1126/stke.2003.167.re2 12554854

[B27] el-Deiry WS WAF1, a potential mediator of p53 tumor suppression Cell 1993 75 4 817 25 10.1016/0092-8674(93)90500-P 8242752

[B28] Gartel AL Radhakrishnan SK Lost in transcription: p21 repression, mechanisms, and consequences Cancer Res 2005 65 10 3980 5 10.1158/0008-5472.CAN-04-3995 15899785

[B29] Prives C Gottifredi V The p21 and PCNA partnership: a new twist for an old plot Cell Cycle 2008 7 24 3840 6 19066467 10.4161/cc.7.24.7243

[B30] Soria G p21 differentially regulates DNA replication and DNA-repair-associated processes after UV irradiation J Cell Sci 2008 121 Pt 19 3271 82 10.1242/jcs.027730 18782865

[B31] Soria G P21Cip1/WAF1 downregulation is required for efficient PCNA ubiquitination after UV irradiation Oncogene 2006 25 20 2829 38 10.1038/sj.onc.1209315 16407842

[B32] Sgambato A Multiple functions of p27(Kip1) and its alterations in tumor cells: a review J Cell Physiol 2000 183 1 18 27 10.1002/(SICI)1097-4652(200004)183:1<18::AID-JCP3>3.0.CO;2-S 10699962

[B33] Zhang X Identification of possible reactive oxygen species involved in ultraviolet radiation-induced oxidative DNA damage Free Radic Biol Med 1997 23 7 980 5 10.1016/S0891-5849(97)00126-3 9358240

[B34] Placzek M Effect of ultraviolet (UV) A, UVB or ionizing radiation on the cell cycle of human melanoma cells Br J Dermatol 2007 156 5 843 7 10.1111/j.1365-2133.2007.07795.x 17355234

[B35] Dankort D Braf(V600E) cooperates with Pten loss to induce metastatic melanoma Nat Genet 2009 41 5 544 52 10.1038/ng.356 19282848 PMC2705918

[B36] Yokoyama S Salma N Fisher DE MITF pathway mutations in melanoma Pigment Cell Melanoma Res 2009 22 4 376 7 10.1111/j.1755-148X.2009.00599.x 19558635

[B37] Cronin JC Frequent mutations in the MITF pathway in melanoma Pigment Cell Melanoma Res 2009 22 4 435 44 10.1111/j.1755-148X.2009.00578.x 19422606 PMC2728363

[B38] Jonsson G Genomic profiling of malignant melanoma using tiling-resolution arrayCGH Oncogene 2007 26 32 4738 48 10.1038/sj.onc.1210252 17260012

[B39] Eisinger M Marko O Selective proliferation of normal human melanocytes in vitro in the presence of phorbol ester and cholera toxin Proc Natl Acad Sci USA 1982 79 6 2018 22 10.1073/pnas.79.6.2018 6952249 PMC346113

[B40] Liu F Lee WH CtIP activates its own and cyclin D1 promoters via the E2F/RB pathway during G1/S progression Mol Cell Biol 2006 26 8 3124 34 10.1128/MCB.26.8.3124-3134.2006 16581787 PMC1446954

